# CDKL2 Is Associated with HER2 Status and Overall Survival in Gastric Cancer: Comparative Analysis of CDKL2 Protein Expression and Gene Copy Number

**DOI:** 10.1155/2020/1712723

**Published:** 2020-10-31

**Authors:** Qiong Shao, Fang Wang, Yuxia Xu, Xu Zhang, Wenting Tang, Yanfen Feng, Yue Li

**Affiliations:** ^1^Sun Yat-sen University Cancer Center, State Key Laboratory of Oncology in South China, Collaborative Innovation Center for Cancer Medicine, Guangzhou 510060, China; ^2^Department of Molecular Diagnostics, Sun Yat-sen University Cancer Center, Guangzhou 510060, China; ^3^Department of Pathology, Sun Yat-sen University Cancer Center, Guangzhou 510060, China

## Abstract

**Background:**

Cyclin-dependent kinase-like 2 (CDKL2) is a member of the CDKL family and recognized as a novel regulator of epithelial-mesenchymal transition of breast cancer cells, but its role has not been explored in gastric cancer (GC). This study was to characterize the CDKL2 protein expression and gene copy number in relation to human epidermal growth factor receptor 2 (HER2) status, clinicopathological features, and overall survival (OS) in GC.

**Methods:**

This study detected the CDKL2 protein expression and gene copy number by immunochemistry (IHC) and fluorescent in situ hybridization (FISH), respectively, in 334 GC samples. HER2 status was determined according to established criteria. Associations of the CDKL2 protein expression and gene copy number with OS in GC were evaluated, and the association between CDKL2 mRNA expression and OS in GC was also analyzed using TCGA data.

**Results:**

The detection results suggested that 34.1% cases showed high CDKL2 protein expression; 11.4% cases had ≥5 copies of CDKL2 gene or a ratio of CDKL2 to chromosome of ≥2. The CDKL2 protein expression was markedly correlated with its gene copy number. High protein expression and high gene copy number were both significantly associated with positive HER2 status, and they both could predicted a shorter OS, although not as independent markers suggested by the multivariate Cox proportional hazard regression analysis. The TCGA data indicated that higher CDKL2 mRNA level also predicted a shorter OS in GC.

**Conclusions:**

The combined detection of the CDKL2 protein level and gene copy number could be of important value in predicting HER2 status and prognosis of patients with GC.

## 1. Introduction

Gastric cancer (GC) is one of the most prevalent digestive system tumors worldwide [1]. Its overall 5-year relative survival rate is about 20% in most areas of the world [2], except in Japan, where the 5-year survival rate of stages I and II patients is above 70% owing to large-scale screening programs [3]. Despite a continued decline in incidence and mortality rates for GC being reported in the past decades, a large global variability still exists geographically [4], and GC remains a major killer across the globe [5]. Currently, solid and pragmatic biomarkers for prognosis of GC patients are limited, except the TNM stage and status of human epidermal growth factor receptor 2 (HER2) which have been found to largely influence GC prognosis and response to therapy [6, 7]. Therefore, more robust prognostic biomarkers are urgently needed to be identified.

Uncontrolled cell cycle leading to persistent cell proliferation is a hallmark of human cancers [8], and cyclin-dependent kinases (CDKs) regulate critical checkpoints of cell cycle [9]. The CDK-like (CDKL) protein kinase family, identified based on their structural relation to CDKs [10], comprises of five members, namely, CDKL1 to CDKL5. Till present, studies exploring the functions of CDKLs in cancers are limited, and most of them are focused on CDKL1. As reported, CDKL1 overexpression could decrease the chemosensitivity of oral squamous cell carcinoma cells to hydroxycamptothecin [11], the reduction of CDKL1 attenuated tumor proliferation and invasion in colorectal cancer [12], inhibited proliferation, and improved apoptosis of GC [13]. Inhibiting CDKL1 could also lead to cell cycle arrest and proliferation hindrance in breast cancer, melanoma, and neuroblastoma [14–16].

CDKL2 was recognized as a novel regulator of epithelial-mesenchymal transition (EMT) and demonstrated to enhance mesenchymal traits and stem-cell like phenotypes of breast cancer cells [17]. However, the roles of CDKL2 protein expression and gene copy number have not been elucidated clearly in GC. The aim of this study was to characterize the CDKL2 protein expression and gene copy number in relation to HER2 status, clinicopathological features, and overall survival (OS) of patients with GC.

## 2. Materials and Methods

### 2.1. Patients

GC paraffin-embedded tissue blocks from 334 patients who underwent surgical resection between November 2011 and December 2012 were collected at the Sun Yat-sen University Cancer Center. These patients had not received any preoperative anticancer therapy. The clinicopathological features of patients including gender, age, TNM stage, Lauren classification, lymph-vascular invasion (LVI), perineural invasion (PNI), and tumor location were retrospectively collected, and their associations with CDKL2 were statistically analyzed. The TNM stage was confirmed as per the eighth edition American Joint Committee on Cancer (AJCC) classification [18]. The OS was defined as the period from surgery to death or to the last follow-up. This study was conducted in accordance with the Helsinki Declaration; the use of patient samples and clinical information was approved by the Ethics Committee of the Sun Yat-sen University Cancer Center.

### 2.2. Immunohistochemistry (IHC) and Hybrid- (H-) Score Evaluation

IHC staining was performed on the basis of a previously established protocol [19]. In brief, the tissue sections were deparaffinized with dimethylbenzene and rehydrated in a series of graded alcohols before being blocked with 0.3% hydrogen peroxide for 15 min. To retrieve antigens, the slides were boiled in tris(hydroxymethyl)aminomethane-EDTA buffer (pH 8.0) in a microwave for 30 min. After blocking by 10% normal goat serum for 20 min to reduce nonspecific staining, the slides were then incubated with monoclonal rabbit anti-CDKL2 antibody (clone OTI5D9, 1 : 150 dilution, Thermo Fisher, Danvers, MA) at 4°C overnight. Next day, the slides were treated with horseradish peroxidase (DAKO ChemMate™ EnVision™ Detection Kit, Copenhagen, Danmark) at 37°C for 30 min and subsequently incubated with the 3,3′-diaminobenzidine (DAB) solution for visualization. At last, nuclear counterstain with freshly diluted hematoxylin was conducted to allow better visualization of the tissue structure.

Two independent observers (XYX and FYF) blinded to the patients' clinicopathological data conducted the H-score assessment for CDKL2 expression. Scoring criteria for staining intensity were as follows: 0 (negative), 1 (weak), 2 (medium), and 3 (strong). The staining extent score was the stained cell percentage of counted cells in 3–5 microscopic fields, ranging from 0 to 100. The final H-score was calculated as 3 × extent score (strong) + 2 × extent score (medium) + 1 × extent score (weak), ranging from 0 to 300.

### 2.3. Fluorescent In Situ Hybridization (FISH) and Interpretative Rules

Briefly, a CDKL2/CEP4 probe cocktail prepared with CDKL2 DNA (RP11-105F22 BAC clone) labeled with SpectrumRed and the SpectrumGreen CEP4 (Vysis; Abbott Laboratories, Chicago, IL, USA). CDKL2 copy number assessment was performed according to a previously published protocol [20]. CDKL2 FISH-positive was defined by the presence of gene copy number ≥ 5 copies per cell or a ratio of CDKL2 gene to chromosome of ≥2.0 in ≥10% of tumor cells. The interpretation of FISH was independently performed by two experts (SQ and ZX).

### 2.4. HER2 Status Assessment

HER2 status was determined in the routine pathological diagnosis. IHC was carried out using a BenchMark XT autostainer (Ventana Medical Systems, Inc.) with monoclonal rabbit anti-HER2/neu antibody (clone 4B5; Ventana Medical Systems, Inc., Tucson, AZ, USA). The protein expression of HER2 was classified according to the widely adopted scoring criteria for GC [21, 22]. The gene copy number of HER2 was assessed by FISH using the HER2 DNA probe kit (PathVysion HER2 DNA Probe Kit, Abbott Laboratories) according to a previously published protocol [20]. The FISH signals were assessed under a microscope (Olympus BX61, Japan) equipped with a triple-pass filter (DAPI/Green/Orange, Vysis). FISH images were acquired using the BioView Automated Imaging Analysis System (BioView Ltd., Rehovot, Israel). The criteria for HER2 amplification were based on Hofmann's criteria in GC [22] that cases with IHC 3+, or IHC 2+ and HER2 amplification by FISH were defined as HER2 positive; other cases were defined as HER2 negative.

### 2.5. Analysis of Prognosis Data from The Cancer Genome Atlas (TCGA)

The CDKL2 mRNA expression information and prognosis data of 378 GC patients were downloaded from OncoLnc website (http://www.oncolnc.org/). This website stores survival data for 8,647 patients from 21 cancer studies performed by TCGA, along with RNA-Seq expression for mRNAs and miRNAs from TCGA. The detailed instruction on how to take the use of functions of this website was elaborated previously [23].

### 2.6. Statistics

The *χ*^2^ test was used to analyze the association between CDKL2 and clinicopathological characteristics of patients with GC. The cut-off value for H-score was evaluated using the receiver operating characteristic (ROC) curve. The OS was evaluated and compared using the Kaplan-Meier method and logrank test. The univariate and multivariate Cox proportional hazard regression analyses were separately performed to identify clinicopathological features that could influence the OS. Only variables reaching a statistical significance in univariate analysis were allowed into the multivariate analysis. *p* < 0.05 based on two-tailed tests was considered statistical significant. All the statistical calculation and graphical representation production were implemented by SPSS 26.0 (IBM Corp., Armonk, NY, USA) and GraphPad Prism 8.0 (San Diego, CA, USA).

## 3. Results

### 3.1. CDKL2 Protein Expression by IHC

**Figure 1:**
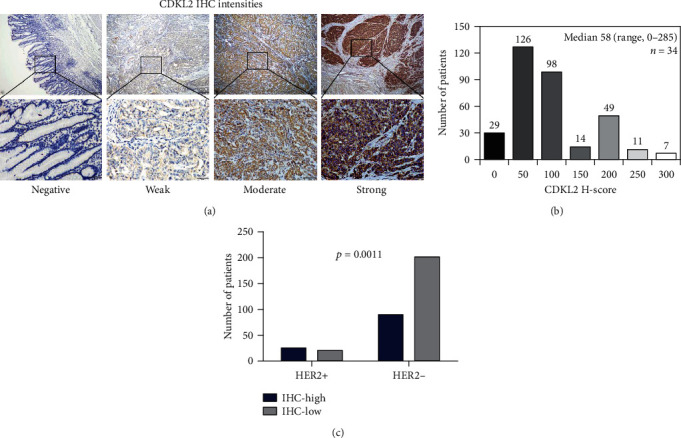
H-score of CDKL2 detected by IHC. (a) Typical images of different staining intensities of CDKL2 by IHC. (b) The patient number in different levels of CDKL2 H-score. (c) The proportion of patients with high CDKL2 protein expression (IHC-high) in the HER2+ group was significantly higher than that in the HER2- group (*p* = 0.0011). Upper panel, ×40, scale bar 200 *μ*m; lower panel, ×200, scale bar 50 *μ*m. IHC: immunohistochemistry.

The pattern of CDKL2 immunostaining was cytoplasmic, and different intensities of IHC staining are shown in Figure 1(a). In total, the median and mean H-score was 58 and 65, respectively, and 264 out of the 334 (79.0%) specimens had a CDKL2 H-score greater than 10. Majority of the cases (67.1%) were within the range of 0 < H–score < 100, 24.3% had H-score higher than 100, and only 8.7% of the cases had negative IHC staining (Figure 1(b)). The ROC determined H-score cut-off point was 67; for this study, cases with H-score of 67 or higher were categorized as the high-expression group and the rest as low-expression group. As shown from the data (Table 1), the proportion of patients with distant metastasis was significantly higher in the high-CDKL2-expression group than in the low-CDKL2-expression group (*p* = 0.049). Also, the ratio of patients with positive HER2 status was markedly higher in the high-CDKL2-expression group than in the low-CDKL2-expression group (*p* = 0.0011, Figure 1(c)), while H-score of CDKL2 seemed not to be connected with other clinicopathological characteristics.

### 3.2. CDKL2 Gene Copy Number Variant by FISH

**Figure 2:**
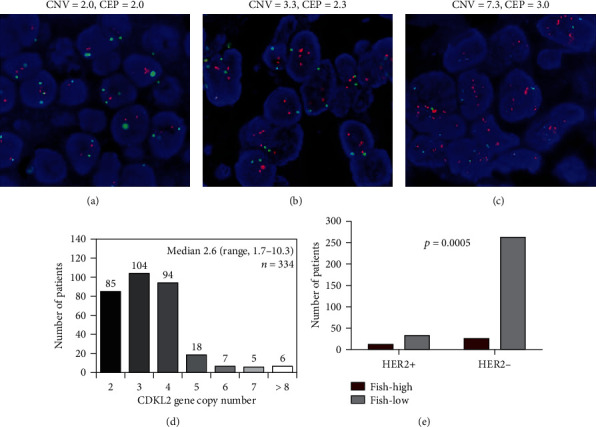
CDKL2 gene copy number detected by FISH. (a–c) Representative images of different CDKL2 gene copy number variants by FISH. (a) Negative FISH result: average CDKL2 copy number was 2.0, the ratio of CDKL2 to chromosome was 1.0. (b) Negative FISH result: average CDKL2 copy number was 3.3, the ratio of CDKL2 to chromosome was 1.4. (c) Positive FISH result: average CDKL2 copy number was 7.3, the ratio of CDKL2 to chromosome was 2.4. (d) The patient number in different levels of CDKL2 gene copy number. (e) The ratio of patients with positive HER2 status was significantly higher in the CDKL2 FISH+ group than in the CDKL2 FISH- group (*p* = 0.0005). FISH: fluorescent in situ hybridization; CNV: copy number variation; CEP: centromeric probe.

Representative images of varying levels of CDKL2 copy numbers are shown in Figures 2(a)–2(c). The average CDKL2 gene copy number per cell was 2.6, ranging from 1.7 to 10.3. It was suggested by the distribution of the copy number that the FISH-positive (FISH+) rate of the entire cohort was 11.4% (38/334), and most patients (78.2%) had <5 copies of CDKL2 (Figure 2(d)). Among the FISH+ samples, one case (0.3%) exhibited CDKL2 gene cluster amplification, 35 (10.5%) had average CDKL2 gene copy number ≥ 5 per nucleus and 10 (3.0%) had a ratio of gene to chromosome ≥2. Statistical analyses indicated that the positive rate of HER2 status in FISH+ patients was much higher than that in FISH- patients (*p* = 0.0005, Table 1 and Figure 2(e)). Other demographic or clinical features were not related to CDKL2 FISH result.

### 3.3. Correlation between CDKL2 Protein Expression and CDKL2 Gene Copy Number

**Figure 3:**
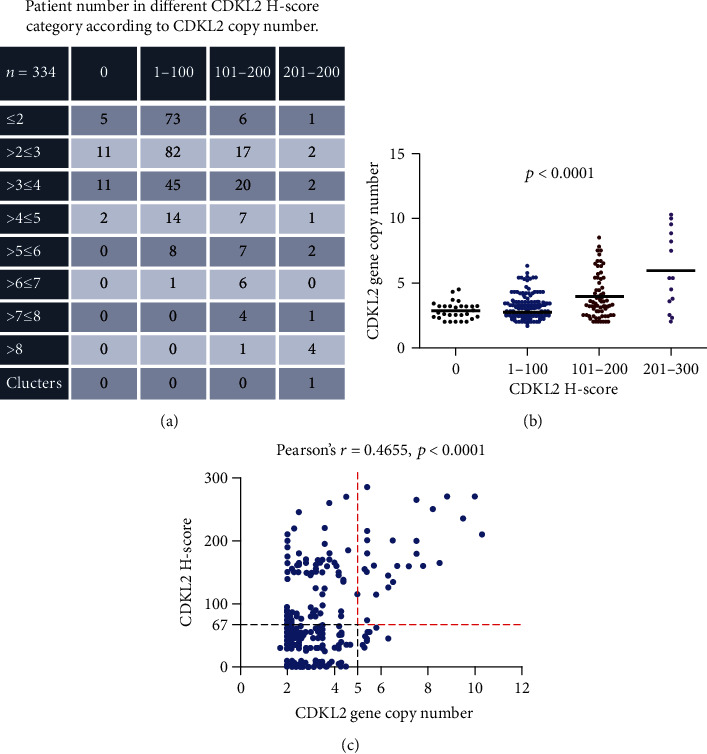
CDKL2 H-score and gene copy number were closely correlated with each other. (a) The number of patients in different CDKL2 H-score category according to CDKL2 gene copy number. (b) The mean CDKL2 gene copy number increased markedly as the H-score category went up (*p* < 0.0001). (c) There was a significant correlation between the CDKL2 H-score and gene copy number (Pearson's *r* = 0.4655, 95%CI = 0.3770–0.5456, *p* < 0.0001).

The number of patients in each H-score category according to the copy number variation was calculated (Figure 3(a)). When the tumors were categorized according to their corresponding CDKL2 H-score of 0, 0–100, 101–200, and 201–300, their median average gene copy number were 2.8, 2.7, 4.0, and 6.0, respectively (*p* < 0.0001, Figure 3(b)). Most patients classified as FISH+ had high CDKL2 H-score; 71.4% (25/35) samples with a gene copy number of 5 or greater expressed higher CDKL2 protein expression (H–score ≥ 67). Moreover, there was a significant correlation between high CDKL2 protein expression and increased CDKL2 copy number (both variables analyzed as continuous data (Pearson's *r* = 0.4655, 95%CI = 0.3770–0.5456, *p* < 0.0001, Figure 3(c)).

### 3.4. Association between CDKL2 and Prognosis

**Figure 4:**
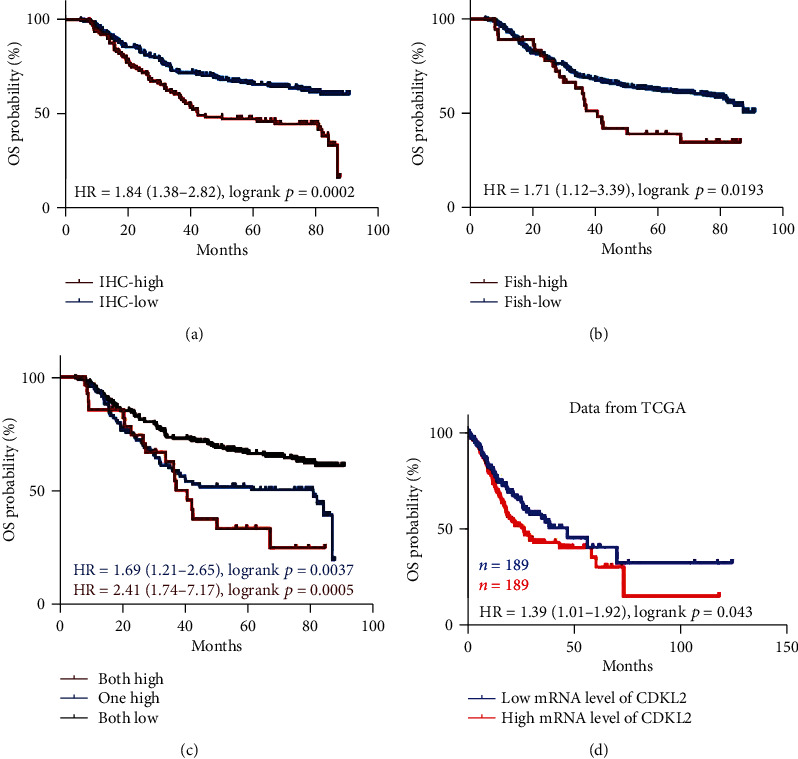
Higher CDKL2 H-score and copy number both predicted worse OS of GC patients. (a) GC patients with higher CDKL2 H-score had a shorter OS than those with lower H-score (HR = 1.84 (1.38–2.82), *p* = 0.0002). (b) GC patients with higher CDKL2 copy number had a worse OS than those with lower CDKL2 copy number (HR = 1.71 (1.12–3.39), *p* = 0.0193). (c) The patients with higher CDKL2 H-score or higher CDKL2 copy number had a poorer OS than those with both lower CDKL2 H-score and lower CDKL2 copy number (HR = 1.69 (1.21–2.65), *p* = 0.0037); the patients with both higher CDKL2 H-score and higher CDKL2 copy number had an even lower OS than those with both lower CDKL2 H-score and lower CDKL2 copy number (HR =2.41 (1.74–7.17), *p* = 0.0005). (d) Data from TCGA showed that GC patients with higher CDKL2 mRNA level had significantly shorter OS than those with lower CDKL2 mRNA level (26.5 vs. 46.9 months, HR = 1.39 (1.01–1.92), *p* = 0.043; the median was set as cut-off point).

High CDKL2 protein expression (IHC+) (42.3 months vs. not arrived, HR = 1.84 (1.38–2.82), *p* = 0.0002; Figure 4(a)) and increased CDKL2 copy number (FISH+) (40.6 months vs. not arrived, HR = 1.71 (1.12–3.39), *p* = 0.0193; Figure 4(b)) both predicted a worse OS of patients with GC compared to those with IHC- and FISH-, respectively. Moreover, IHC+FISH-/IHC-FISH+ patients (80.7 months vs. not arrived, HR = 1.69 (1.21–2.65), *p* = 0.0037) and IHC+FISH+ patients (40.6 months vs. not arrived, HR = 2.41 (1.74–7.17), *p* = 0.0005; Figure 4(c)) had significant shorter OS compared to IHC-FISH- patients.

In univariate analysis, CDKL2 H-score, CDKL2 gene copy number, age, T stage, N stage, M stage, TNM stage, LVI, PNI, and HER2 status were found to be significantly associated with OS. Among them, only age, T stage, M stage, TNM stage, LVI, and HER2 status remained significantly influential for OS in the multivariate analysis, indicating that higher CDKL2 protein expression and copy number could have an adverse influence on the OS of GC patients, but they seemed not to act as independent biomarkers (Table 2).

To further validate the prognostic role of CDKL2, we explored the influence of CDKL2 mRNA expression on the OS of GC patients using data from TCGA. The result showed that GC patients with higher CDKL2 mRNA level had significantly shorter OS than those with lower CDKL2 mRNA level (26.5 vs. 46.9 months, HR = 1.39 (1.01–1.92), *p* = 0.043, the median was set as a cut-off point, Figure 4(d)), which was consistent with the protein expression and gene copy number results in our analysis.

## 4. Discussion

In the present study, we explored the CDKL2 protein expression and gene copy number using IHC and FISH, respectively, and also evaluated their associations with clinicopathological parameters in GC patients. We found that 34.1% of the patients expressed a high level of CDKL2 protein and 11.4% had increased CDKL2 copy numbers. Patients with a high expression level of CDKL2 protein tended to have an increased CDKL2 copy number. Moreover, a high level of protein expression or a high gene copy number of CDKL2 was significantly associated with positive HER2 status and worse OS.

CDKL2 H-score and CDKL2 gene copy number both could predict shorter OS of GC patients in the univariate analysis, and their predictable value for OS would increase a lot when they were analyzed jointly. However, they were not independent prognostic factors for OS as suggested by the multivariate analysis, which might be due to their tight relation with HER2 status, a very robust biomarker for targeted therapy efficacy and prognosis of GC as shown by previous research [6, 24]. We speculated that the CDKL2 protein expression or gene copy number could influence OS of GC patients not by itself but by cooperating with other hub biomarkers such as HER2 status and distant metastasis.

HER2 is a protooncogene located in 17q21. HER2 overexpression or amplification is found in 6–30% GC cases and indicates therapeutic effects of targeted drugs [25]. Our statistical analyses indicated a close association between CDKL2 and HER2 status, but the mechanism behind this correlation is unclear. According to the previous reports, CDKL2 is a newly discovered regulator enhancing EMT and increasing CD44-high subpopulation through upregulating ZEB1 expression in breast cancer [17]. And that the promoting effect of TGF-*β* on EMT could be potentiated by oncogene HER2, epidermal growth factor (EGF), or MEK5-ERK signaling in breast cancer [26]. Therefore, CDKL2 and HER2 might exert important effects on EMT and stemness of malignant cells jointly. In addition, the phosphorylation of GST-Myc induced by CDKL2 (p56 or KKIAMRE) significantly increased when treated with 100 nM EGF for 0-60 min, indicating that CDKL2 could be bound and activated by EGF, the well-established ligand for HER2 [27]. Hence, there might be some crosstalk existing between CDKL2-related and HER2-related signaling, which warrants further experimental exploration in GC.

EMT has been widely recognized as a key process contributing to GC progression, where cancer cells go through phenotypic alterations and thus acquiring the potential to migrate and infiltrate. In addition to classical EMT markers, such as E-cadherin, *β*-catenin, and vimentin, many protein kinases have been reported to promote EMT including CDKL2 [28], also known as p56 or KKIAMRE; it belongs to a cdc2-related serine/threonine protein kinase family [29, 30] and was reported to induce EMT, tumor formation, and metastasis by activating a ZEB1/E-cadherin/*β*-catenin-positive feedback loop in several human breast cell lines and in orthotopic breast cancer xenograft model [17], which supported an oncogenic role of CDKL2 and was consistent with our results. It might be of value to jointly detect the CDKL2 and EMT markers in the future study to better predict the prognosis of patients and understand the association of CDKL2 copy number with EMT in GC.

Recently, a study by Fang et al. also performed in GC claimed that CDKL2 could impair cell growth and invasion and that patients with low CDKL2 expression had significantly poorer disease-free survival and OS compared with those with high CDKL2 expression [31]. By contrast, our results are in line with TCGA data that GC patients with high CDKL2 mRNA level had significantly shorter OS than those with low CDKL2 mRNA level. In addition, the other members in the CDKL family, such as CDKL1, CDKL3, and CDKL4, also exhibited oncogenic effect in different malignancies [11–16, 32, 33]. The possible causes for the discrepant conclusions between Fang et al.'s study and the others may be the different sample sizes and different interpretation standards for IHC result [31]. To be specific, Fang et al. included 151 GC samples into their research and judged the IHC result according to only the staining extent, while we collected surgical samples from 334 patients with GC and decided the IHC H-score considering both staining extent and staining intensity. To deal with the heterogeneity of staining, different staining intensity scores were weighted by their corresponding extent scores when calculating the H-score in our study. To sum up, the contradicted results of CDKL2 by different studies suggested that the role of CDKL2 expression in human cancers is more complicated than expected and warrants further deep research.

We found that there was a significant correlation between the CDKL2 protein expression and CDKL2 gene copy number; this result could be a useful support to establish the interpretation criteria similar with that of HER2 for CDKL2; that is, samples with equivocal IHC results need to be further validated by FISH [34]. Another example is MET, a well-known drug target in non-small-cell lung cancer, both protein overexpression and gene amplification of MET indicated a better response of patients to MET inhibitor treatment and worse prognosis [35–37]. Therefore, our study provides preliminary data depicting CDKL2 dosage in different molecular layers in GC and indicated it has a potential to serve as a drug target and prognostic biomarker for GC patients.

The present study has a couple of strengths and limitations. This study represents the first combined analysis of CDKL2 protein expression and gene copy number; additionally, TCGA data of CDKL2 mRNA level was also analyzed, providing more information for the role of CDKL2 in GC from three different molecular layers. What is more, we detected the HER2 status for each sample as per well-defined procedure and spot the tight association between CDKL2 and HER2 status. Since both IHC and FISH methods are widely applicable to clinical specimens in most hospitals, domestic and overseas, detection of CDKL2 is of potential application value in clinical practice. Here, we should acknowledge some limitations. Due to the lack of lifestyle information, we could not include factors like smoking status or Helicobacter pylori infection that may affect GC progression into our analysis. The biological effect and possible molecular mechanism of CDKL2 in GC were not explored by experiments and remained to be elucidated by further research.

## 5. Conclusion

In this study, we found that CDKL2 protein expression was closely correlated with its copy number; both of them were tightly correlated with HER2 status and predicted a worse OS of GC patients, indicating that CDKL2 might have an oncogenic role in GC and was of important value in predicting HER2 status and prognosis of patients with GC.

## Figures and Tables

**Table 1:** Patient characteristics according to the CDKL2 expression by IHC and FISH.

		All patients		CDKL2-IHC low		CDKL2-IHC high			CDKL2-FISH low		CDKL2-FISH high		
Variable		No.	%	No.	%	No.	%	*p*	No.	%	No.	%	*p*
Gender	Male	228	68.3	149	65.4	79	34.6	0.770	203	89.0	25	11.0	0.087
	Female	106	31.7	71	67.0	35	33.0		93	87.7	13	12.3	
Age	>60	155	46.4	101	65.2	54	34.8	0.800	135	87.1	20	12.9	0.414
	≤60	179	53.6	119	66.5	60	33.5		161	89.9	18	10.1	
Histologic grade	Moderate	51	15.3	34	66.7	17	33.3	0.896	44	86.3	7	13.7	0.566
	Poor	283	84.7	186	65.7	97	34.3		252	89.0	31	11.0	
TNM^a^	1-2	141	42.2	99	70.2	42	29.8	0.152	124	87.9	17	12.1	0.738
	3-4	193	57.8	121	62.7	72	37.3		172	89.1	21	10.9	
Lauren classification	Intestinal	102	30.5	67	65.7	35	34.3	0.805	86	84.3	16	15.7	0.158
	Diffuse	167	50.0	108	64.7	59	35.3		149	89.2	18	10.8	
	Mixed	65	19.5	45	69.2	20	30.8		61	93.8	4	6.2	
T	1-2	83	24.9	54	65.1	29	34.9	0.858	74	89.2	9	10.8	0.860
	3-4	251	75.1	166	66.1	85	33.9		222	88.4	29	11.6	
N	N0	97	29.0	63	64.9	34	35.1	0.821	89	91.8	8	8.2	0.249
	N1-3	237	71.0	157	66.2	80	33.8		207	87.3	30	12.7	
M	M-	292	86.8	198	67.9	94	32.1	0.049	259	88.7	33	11.3	0.908
	M+	42	13.2	22	52.4	20	47.6		37	88.1	5	11.9	
LVI	+	122	36.5	76	62.3	46	37.7	0.296	109	89.3	13	10.7	0.753
	-	212	63.5	144	67.9	68	32.1		187	88.2	25	11.8	
PNI	+	194	58.1	121	62.4	73	37.6	0.113	169	87.1	25	12.9	0.306
	-	140	41.9	99	70.7	41	29.3		127	90.7	13	9.3	
Tumor location	Cardia	54	16.2	36	66.7	18	33.3	0.892	50	94.4	4	5.6	0.316
	Other	280	83.8	184	65.7	96	34.3		246	87.9	34	12.1	
HER2 status	+	45	13.5	20	44.4	25	55.6	0.001	33	73.3	12	26.7	0.001
	-	289	86.5	200	69.2	89	30.8		263	91.0	26	9.0	

^a^Clinical stage were reclassified according to the 8th American Joint Committee on Cancer (AJCC) TNM classification. LVI: lymph-vascular invasion; PNI: perineural invasion.

**Table 2:** Cox proportional hazard model analysis of prognostic factors.

Variables	Univariate analysis			Multivariate analysis		
	HR	95% CI	*p*	HR	95% CI	*p*
CDKL2 H-score (high vs. low)	1.848	1.325-2.576	<0.001	1.322	0.894-1.954	0.162
CDKL2 gene copy number (high vs. low)	1.801	1.151-2.818	0.010	1.160	0.694-1.938	0.571
Sex (male vs. female)	1.023	0.720-1.388	0.1453	—	—	—
Age (≥60 vs. <60 years)	1.809	1.299-2.518	<0.001	1.695	1.172-2.452	0.005
Depth of invasion (T3/T4 vs. T1/T2)	3.518	2.090-5.922	<0.001	2.024	1.021-4.012	0.043
Lymph node metastasis (N3 vs. other)	2.717	1.954-3.779	<0.001	1.270	0.831-1.940	0.270
Distant metastasis (M1 vs. other)	3.154	2.113-4.709	<0.001	1.742	1.104-2.748	0.017
TNM stage (III + IV vs. I + II)	3.035	2.077-4.433	<0.001	4.350	2.297-8.238	<0.001
Differentiation (poor vs. moderate)	1.045	0.663-1.645	0.851	—	—	—
Lauren classification (intestinal vs. diffuse vs. mixed)	1.014	0.709-1.450	0.940	—	—	—
Lymph-vascular invasion (positive vs. negative)	2.519	1.812-3.501	<0.001	1.543	1.047-2.275	0.028
Perineural invasion (positive vs. negative)	2.593	1.788-3.762	<0.001	1.555	0.992-2.436	0.054
HER2 status (positive vs. negative)	3.040	2.051-4.505	<0.001	2.449	1.611-3.722	<0.001
Location (cardia vs. other)	1.402	0.921-2.132	0.115	—	—	—

IHC: immunohistochemical; HR: hazard ratio; CI: confidence interval; *p* < 0.05 was considered statistically significant.

## Data Availability

The data used to support the findings of this study are available from the corresponding author upon request.

## Abbreviations

AJCC: American Joint Committee on Cancer
CDKs: Cyclin-dependent kinase
CDKL2: Cyclin-dependent kinase-like 2
CI: Confidence interval
EGF: Epidermal growth factor
EMT: Epithelial-mesenchymal transition
FISH: Fluorescent in situ hybridization
GC: Gastric cancer
HER2: Human epidermal growth factor receptor 2
HR: Hazard ratio
H-score: Hybrid-score
IHC: Immunohistochemistry
LVI: Lymph-vascular invasion
PNI: Perineural invasion
OS: Overall survival
ROC: Receiver operating characteristic
TCGA: The Cancer Genome Atlas.
